# Sent packing: protein engineering generates a new crystal form of *Pseudomonas aeruginosa* DsbA1 with increased catalytic surface accessibility

**DOI:** 10.1107/S1399004715018519

**Published:** 2015-11-26

**Authors:** Roisin M. McMahon, Mathieu Coinçon, Stephanie Tay, Begoña Heras, Craig J. Morton, Martin J. Scanlon, Jennifer L. Martin

**Affiliations:** a Institute for Molecular Bioscience, Division of Chemistry and Structural Biology, University of Queensland, 306 Carmody Road, Brisbane, Queensland 4072, Australia; b Biota Holdings Limited, Unit 10, 585 Blackburn Road, Notting Hill, Victoria 3168, Australia; c Medicinal Chemistry, Monash Institute of Pharmaceutical Sciences, Monash University, 381 Royal Parade, Parkville, Victoria 3052, Australia

**Keywords:** bacterial virulence, drug design, fragment-based lead discovery, DsbA

## Abstract

*Pseudomonas aeruginosa* is an opportunistic human pathogen for which new antimicrobial drug options are urgently sought. *P. aeruginosa* disulfide-bond protein A1 (PaDsbA1) plays a pivotal role in catalyzing the oxidative folding of multiple virulence proteins and as such holds great promise as a drug target. As part of a fragment-based lead discovery approach to PaDsbA1 inhibitor development, the identification of a crystal form of PaDsbA1 that was more suitable for fragment-soaking experiments was sought. A previously identified crystallization condition for this protein was unsuitable, as in this crystal form of PaDsbA1 the active-site surface loops are engaged in the crystal packing, occluding access to the target site. A single residue involved in crystal-packing interactions was substituted with an amino acid commonly found at this position in closely related enzymes, and this variant was successfully used to generate a new crystal form of PaDsbA1 in which the active-site surface is more accessible for soaking experiments. The PaDsbA1 variant displays identical redox character and *in vitro* activity to wild-type PaDsbA1 and is structurally highly similar. Two crystal structures of the PaDsbA1 variant were determined in complex with small molecules bound to the protein active site. These small molecules (MES, glycerol and ethylene glycol) were derived from the crystallization or cryoprotectant solutions and provide a proof of principle that the reported crystal form will be amenable to co-crystallization and soaking with small molecules designed to target the protein active-site surface.

## Abstract 2

The crystal structure of a *P. aeruginosa* DsbA1 variant is more suitable for fragment-based lead discovery efforts to identify inhibitors of this antimicrobial drug target. In the reported structures the active site of the protein can simultaneously bind multiple ligands introduced in the crystallization solution or *via* soaking.

## 1. Introduction   

Disulfide-bond (DSB) proteins are bacterial oxidoreductase enzymes that catalyze the folding of disulfide bonds in unfolded or partially folded protein substrates. The primary oxidase is DsbA, which typically employs a Cys-Xaa-Xaa-Cys (C*XX*C) active-site motif to catalyze a series of thiol-exchange reactions that ultimately oxidize reduced protein substrates. DsbA is itself maintained in an active state by interactions with a partner redox membrane protein (DsbB). DsbA substrates include many structural and secreted virulence effector proteins, and there is now overwhelming evidence that the DSB machinery plays a vital role in the pathogenicity of Proteobacteria (Heras *et al.*, 2009 ▸); deletion of DsbA prevents lethal bacterial infection *in vivo* and has pleiotropic effects upon virulence (Ireland *et al.*, 2014 ▸; Totsika *et al.*, 2009 ▸). In the multidrug-resistant and opportunistic Gram-negative bacterium *Pseudomonas aeruginosa*, deletion of DsbA impairs the secretion and activity of a number of virulence effector proteins (Braun *et al.*, 2001 ▸; Dacheux *et al.*, 2002 ▸; Ha *et al.*, 2003 ▸) and disrupts bacterial motility (Ha *et al.*, 2003 ▸; Urban *et al.*, 2001 ▸). As a consequence of its role in the activity of multiple downstream effector proteins, *P. aeruginosa* DsbA (PaDsbA1) is a promising target for inhibition and hence novel antimicrobial development.

Fragment-based lead discovery (FBLD) approaches offer an efficient way to identify small-molecule binders that can subsequently be elaborated or linked to generate eventual drug-like compounds (Erlanson, 2012 ▸). Indeed, the approach has recently led to the design of early-stage inhibitors of *Escherichia coli* DsbA (Adams *et al.*, 2015 ▸). Highly advantageous for the iterative progression from hit to lead in such an FBLD campaign is high-resolution structural information about target–compound interactions to inform rational medicinal chemistry efforts.

The crystal structure of PaDsbA1 has previously been determined to 1.5 Å resolution (Shouldice *et al.*, 2010 ▸). However, inspection of the crystal packing revealed that a network of interactions, in particular a salt bridge, engages the active-site surface in a tightly packed crystal lattice inter­action. This interaction may occlude access of ligands to the active site in crystal-soaking experiments, thereby impeding the determination of protein–fragment complex crystals; indeed, our efforts to use this crystal form for fragment-soaking experiments did not result in any protein–fragment structures (data not shown). To break this salt bridge, we replaced a single amino acid (Glu82) with a residue commonly found at this position in closely related enzymes and obtained crystals in a new space group with altered crystal packing and increased solvent-channel access to the target site. This variant of PaDsbA1 is biochemically and structurally very similar to wild-type PaDsbA1, with conformational changes confined to the N-terminal end of helix 3. Furthermore, structures of the PaDsbA1 variant were determined in complex with multiple small molecules bound on its active-site surface, one from the crystallization condition and two from cryoprotectant solutions, providing proof of principle that in the new crystal form the active-site face is accessible to compounds introduced *via* soaking or co-crystallization.

## 2. Methods   

### 2.1. Expression-vector construction   

The *P. aeruginosa* *dsbA* gene lacking a signal sequence was previously inserted into a modified pET-28a plasmid (Novagen) to generate a construct with a *Tobacco etch virus* (TEV) protease-cleavable N-terminal His_6_ tag (Shouldice *et al.*, 2010 ▸).

### 2.2. Site-directed mutagenesis   

Glu82 of PaDsbA1 forms an ionic interaction with an active-site residue in the reported crystal structure. The amino-acid sequence of PaDsbA1 (UniProt ID P0C2B2) was aligned with homologues from *E. coli*, *Klebsiella pneumoniae* and *Vibrio cholerae* using *ClustalW*2 to identify residues to replace Glu82. Inspection of the resulting alignment identified that hydrophobic aliphatic side chains (Ile and Met) were found at positions equivalent to Glu82 of PaDsbA1. Subsequently, a single point mutation (Glu82 to Ile, hereafter referred to as E82I PaDsbA1) was introduced using a site-directed mutagenesis approach. Two complementary primers (5′-CAG ATG TTT CTG ACA CTG ATA AGT ATG GGC GTA GAA C-3′ and 5′-GTT CTA CGC CCA TAC TTA TCA GTG TCA GAA ACA TCT G-3′) containing the desired isoleucine mutation and designed to anneal to opposite strands of the template vector were synthesized (GeneWorks, Australia). A 25 µl reaction containing 10 ng double-stranded parental vector, 5 µ*M* of each primer, 1 m*M* dNTP and 0.5 µl *PfuTurbo* DNA polymerase (Pacific Laboratory Products, Australia) was subjected to 25 thermal cycles (95°C for 50 s, 56°C for 60 s and 68°C for 9 min) with a final extension of 10 min at 68°C. The parental vector was subsequently digested with 20 units of DpnI (New England BioLabs) at 37°C for 1 h and the resulting product was used to transform competent TOP10 *E. coli* cells. The introduced mutation was confirmed by sequencing (Australian Genome Research Facility, Australia).

### 2.3. Protein expression and purification   

Wild-type (WT) PaDsbA1 (212 amino acids, 23.7 kDa) and E82I PaDsbA1 (212 amino acids, 23.7 kDa) were expressed and purified largely as described in Shouldice *et al.* (2010 ▸), but with some modifications. Expression was performed in *E. coli* BL21 (DE3) pLysS cells at 30°C and 200 rev min^−1^ for 18 h in ZYP-5052 autoinduction medium [10%(*w*/*v*) tryptone, 5%(*w*/*v*) yeast extract, 0.5%(*v*/*v*) glycerol, 0.05%(*w*/*v*) glucose, 0.2%(*w*/*v*) α-lactose, 25 m*M* ammonium sulfate, 50 m*M* KH_2_PO_4_, 50 m*M* Na_2_HPO_4_, 1 m*M* magnesium sulfate and trace metals] in the presence of ampicillin (100 µg ml^−1^) and chloramphenicol (34 µg ml^−1^). Harvested cells were resuspended in a solution consisting of 25 m*M* Tris pH 7.5, 150 m*M* NaCl, DNAse (Roche Diagnostics, Australia) and protease inhibitors (BioPioneer Inc., California, USA) and were lysed using a Constant Systems Cell Disrupter (single passage at 124 MPa pressure), and the soluble cell fraction was isolated by centrifugation (40 000*g* for 30 min). Immobilized metal-affinity chromatography (IMAC) purification was performed in batch using TALON resin (Clontech) equilibrated in 25 m*M* HEPES pH 7.4, 150 m*M* NaCl, 2 m*M* imidazole. Following wash steps (25 m*M* HEPES pH 7.4, 500 m*M* NaCl, 10 m*M* imidazole), purified protein was eluted with 25 m*M* HEPES pH 7.4, 150 m*M* NaCl, 200 m*M* imidazole. Purified protein was buffer-exchanged on a Sepharose gel-filtration column to remove imidazole prior to cleavage of the N-terminal His_6_ tag by treatment with His-tagged TEV protease at a mass ratio of 1:100 enzyme:substrate. Contaminating TEV protease and uncleaved PaDsbA1 were removed by a second IMAC step. Purified tag-free PaDsbA1 (192 amino acids, 21.3 kDa for both WT and E82I PaDsbA1) was oxidized with a 50:1 molar excess of oxidized glutathione (Sigma–Aldrich). Finally, protein monodispersity and purity (>95%) were confirmed by size-exclusion chromatography (Superdex 75 16/60, GE Healthcare Life Sciences) and SDS–PAGE analysis, respectively. The protein oxidation state was confirmed by an Ellman assay (Ellman *et al.*, 1961 ▸).

### 2.4. Screening, optimization and routine production of E82I PaDsbA1 crystals   

For all experiments, E82I PaDsbA1 was crystallized using the UQ ROCX Facility, the vapour-diffusion method and hanging drops. 96-well plates (100 nl protein solution and 100 nl reservoir solution equilibrated against 75 µl reservoir solution) were set up using a Mosquito crystallization robot (TTP Labtech) against commercially available crystallization screens or bespoke crystallization formulations prepared using a Tecan liquid-handling robot and the *RockMaker* software (Formulatrix). All crystallization experiments were maintained at 20°C. Screening of commercially available crystallization screens with PaDsbA1 at a concentration of 57 mg ml^−1^ identified an initial condition [25%(*w*/*v*) PEG 1500, 0.1 *M* MMT pH 6.0; PACT (Qiagen) condition No. 39] that yielded single crystals from spontaneous nucleation. Subsequent optimization efforts demonstrated that single crystals could be obtained in 20–29%(*w*/*v*) PEG 1500, 0.1 *M* MES pH 6.0, but the drops often required microseeding if spontaneous nucleation did not occur within 2–3 d. Crystals were cuboid in shape and typically 60–170 µm (length) × 30–60 µm (width) × 20–40 µm (depth) in size.

### 2.5. X-ray data collection   

To cryocool the crystals, cryoprotectant solutions were prepared from stock solutions such that they matched the concentration of the mother-liquor components with the addition of either 40% polyethylene glycol 400, 20% ethylene glycol or 20% glycerol. In each case, crystals were briefly (<30 s) soaked in the respective cryoprotectant solution prior to plunging them into liquid nitrogen. X-ray data were collected at 100 K on the microfocus beamline MX2 at the Australian Synchrotron using an ADSC Quantum 315r detector. Data were collected at a wavelength of 0.95370 Å over a total angular rotation of 180° using 1° φ slices with an exposure time of 1 s. The data were indexed and integrated with *iMosflm* (Battye *et al.*, 2011 ▸) and scaled using *POINTLESS* and *SCALA* or *AIMLESS* (Evans, 2006 ▸, 2011 ▸; Evans & Murshudov, 2013 ▸) within the *CCP*4 suite (Winn *et al.*, 2011 ▸). For crystals 1 and 2, all collected data were used and no limit on resolution was imposed during processing. For crystal 3, a resolution limit of 1.7 Å was imposed to maintain a mean *I*/σ(*I*) in the highest resolution shell close to 2.

### 2.6. Structure determination and refinement   

All phasing and model-refinement procedures were implemented within the *PHENIX* software suite. The data were phased using molecular-replacement methods with *AutoMR* (McCoy *et al.*, 2007 ▸) using WT PaDsbA1 (PDB entry 3h93; Shouldice *et al.*, 2010 ▸) as a search model. The resulting model was subjected to iterative rounds of refinement including refinement using TLS groups and refinement of H atoms using a riding model (*phenix.refine*; Afonine *et al.*, 2012 ▸) and model building using *Coot* (Emsley & Cowtan, 2004 ▸). Nonwater atoms were refined with anisotropic atomic displacement parameters. Coordinates and restraints for PEG 400, ethylene glycol, glycerol and MES molecules were generated using *elBOW* (Moriarty *et al.*, 2009 ▸) and the quality of the final model was assessed with *MolProbity* (Chen *et al.*, 2010 ▸) throughout the refinement process. Data-collection and refinement statistics are summarized in Tables 1 ▸ and 2 ▸. The final refined structures have been deposited in the Protein Data Bank (PDB entries 4zl7, 4zl8 and 4zl9). All structural figures were generated using *PyMOL* (v.1.6; Schrödinger; http://pymol.org) and *Adobe Illustrator*.

### 2.7. Redox potential determination   

The redox potentials of WT and E82I PaDsbA1 were determined fluorometrically by exploiting the redox-state-dependent changes in the intrinsic fluorescence of a tryptophan residue close to the catalytic disulfide bridge. Oxidized PaDsbA1 samples were prepared by incubation with a molar excess of oxidized glutathione, which was subsequently removed using an NAP-5 desalting column (GE Healthcare). The redox state of the protein was confirmed by an Ellman assay (Ellman *et al.*, 1961 ▸). Oxidized PaDsbA1 (2 µ*M*) in 100 m*M* sodium phosphate pH 7.0, 0.1 m*M* EDTA was combined with oxidized glutathione (1 m*M*) and a range of concentrations of reduced glutathione (4 µ*M* to 100 m*M*) and allowed to equilibrate at 30°C overnight. The fraction of reduced and oxidized PaDsbA1 at equilibrium was determined from the relative fluorescence intensity of each reaction (excitation at 280 nm, emission at 340 nm) recorded in a black 96-well OptiPlate (Perkin­Elmer, Australia) using a Synergy H1 multimode plate reader (Biotek, Millennium Science, Australia). Six independent experiments were performed to determine the equilibrium constant with glutathione. The redox potential was calculated using the Nernst equation as described previously (Wunderlich & Glockshuber, 1993 ▸).

### 2.8. Insulin reduction   

The protein disulfide reductase activity of WT and E82I PaDsbA1 was measured *in vitro* using an insulin-reduction assay (Holmgren, 1979 ▸). Briefly, in this reaction DsbA catalyzes the reduction (in the presence of DTT) of the intermolecular disulfide bonds between chains A and B of insulin. Upon reduction of the disulfide bonds, chain B precipitates, which can be detected as an increase in the optical density at 650 nm. Reaction solutions were prepared in a final volume of 200 µl in a 96-well transparent clear-bottomed plate and *A*_650 nm_ measurements were made at 15 s intervals over a period of 80 min on a Synergy H1 multimode plate reader (Biotek, Millennium Science, Australia). Reduced WT or E82I PaDsbA1 was prepared at a final concentration of 10 µ*M* in 100 m*M* sodium phosphate pH 7.0, 2 m*M* EDTA, 0.3 m*M* DTT. Insulin (human insulin; Sigma–Aldrich) was added immediately prior to measurements at a final concentration of 0.131 m*M*.

### 2.9. Model peptide-substrate folding assay   

The oxidase activity of WT and E82I PaDsbA1 was assayed using a peptide-oxidation assay, in which the steady-state fluorescence of a substrate peptide is directly coupled to its oxidation state. The substrate is dually labelled with a lanthanide metal ion (europium) and a sensitizer (methylcoumarin amide; MCA), which are spatially distant from one another in the reduced state but are in sufficiently close proximity to support efficient energy transfer in the oxidized state. Oxidation of the peptide substrate can be followed by measuring the fluorescence emission intensity of europium after specific MCA excitation. Accordingly, 80 n*M* WT or E82I PaDsbA1 in 50 m*M* MES pH 5.5, 50 m*M* NaCl, 2 m*M* EDTA was combined with 2 m*M* oxidized glutathione and 8 µ*M* peptide substrate in a total volume of 50 µl. Oxidation of the peptide substrate was monitored in a Synergy H1 multimode plate reader using 384-well white OptiPlates (PerkinElmer, Australia) with excitation at 340 nm and emission at 615 nm, a 150 ms delay before reading and a 100 ms reading time.

## 3. Results and discussion   

### 3.1. Protein engineering disrupts an active-site-mediated crystal-packing interaction   

The active-site surface of DsbA enzymes is composed of a C*XX*C motif located at the N-terminal end of H1 in the thioredoxin domain and three additional loop regions (L1, linking β3 and H2; L2, linking H6 and β4 (the *cis*-Pro loop of the thioredoxin fold; Martin *et al.*, 1993 ▸); and L3, linking β5 and H7, described in McMahon *et al.*, 2014 ▸), which together generate the redox-active site of the enzyme and form the surface for engagement with substrate and partner proteins.

Inspection of the crystal packing of the 1.5 Å resolution WT PaDsbA1 (Shouldice *et al.*, 2010 ▸; PDB entry 3h93) crystal structure revealed that a number of intermolecular inter­actions engage the active-site surface in crystal lattice contacts; in particular, a salt bridge (2.88 Å) between His39 of the active-site Cys-His-Pro-Cys motif and Glu82 of a symmetry-related mate and a hydrogen-bonding interaction (3.25 Å) between the backbone N atom of Val153 (L3) and the carboxyl O atom of a Ser83 symmetry mate. Together, these result in a surface packing that may impede small-molecule access to the active site during crystal soaking experiments. Screening of alternative crystallization conditions using WT PaDsbA1 yielded crystals of different space groups (*P*4 and *P*4_1_ with two molecules in the asymmetric unit) but did not break the primary His39–Glu82_sym_ crystal contact (data not shown).

**Figure 1:**
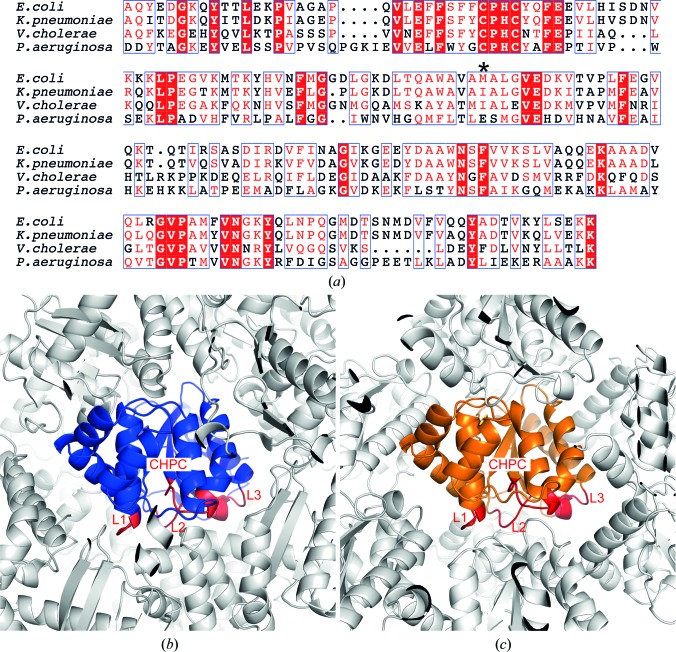
Site-directed mutagenesis disrupts crystal packing involving active-site surface loops. (*a*) Sequence alignment of *P. aeruginosa* DsbA1 with homologues from *E. coli*, *K. pneumoniae* and *V. cholerae* to identify a suitable nonpolar amino-acid substitution for Glu82 in *P. aeruginosa* (marked with an asterisk). The figure was generated using *ESPript*3 (Robert & Gouet, 2014 ▸). Comparison of symmetry-related molecule packing in crystals of WT PaDsbA1 (*b*) (blue) and E82I PaDsbA1 (*c*) (orange) indicates reduced involvement of the active-site surface loops (shown in red for each protein) in packing interactions in E82I PaDsbA1 relative to WT PaDsbA1.

To break the critical salt bridge, we employed site-directed mutagenesis, replacing Glu82 with isoleucine. We selected isoleucine for two reasons: firstly, its uncharged side chain cannot engage in electrostatic contacts and, secondly, isoleucine is found at this position in the closely related enzymes *V. cholerae* DsbA and *K. pneumoniae* DsbA (Fig. 1 ▸ *a*) and substitution was therefore anticipated to have a minimal effect on both protein structure and function. We subsequently obtained crystals of the E82I PaDsbA1 variant in a new space group (*P*2_1_, one molecule in the asymmetric unit). The structure of the E82I PaDsbA1 variant (crystal 1) was determined to 1.9 Å resolution with an *R* factor of 15.9% (*R*_free_ of 20.2%), indicating that the model represents the data with high accuracy. Details of data collection, solution methods and additional indicators of the quality of the final model are given in Tables 1 ▸ and 2 ▸. Inspection of the structure confirmed that our site-directed mutagenesis approach successfully disrupted the previous intermolecular salt bridge. Calculation of the total surface area of interfacing surfaces involved in intermolecular crystal interactions using the *Protein Interfaces, Surfaces and Assemblies* service *PISA* at the European Bioinformatics Institute (http://www.ebi.ac.uk/pdbe/prot_int/pistart.html) found that relative to WT PaDsbA1, E82I PaDsbA has a ∼20% reduction in its total crystal-packing interaction surface area (1238 Å^2^ for WT PaDsbA1 compared with 1536 Å^2^ for WT PaDsbA1; Krissinel & Henrick, 2007 ▸). In the new packing configuration, no protein interactions are formed to His39 (Table 3 ▸). Furthermore, relative to the WT PaDsbA1 crystals, fewer atoms of the active-site surface loops of the E82I PaDsbA1 variant are engaged in crystal contacts with protein molecules of neighbouring symmetry-related molecules (Figs. 1 ▸ *b* and 1 ▸ *c*, Table 3 ▸).

### 3.2. The E82I substitution induces a localized conformational change at the N-terminal end of helix 3 but does not affect the active-site surface   

**Figure 2:**
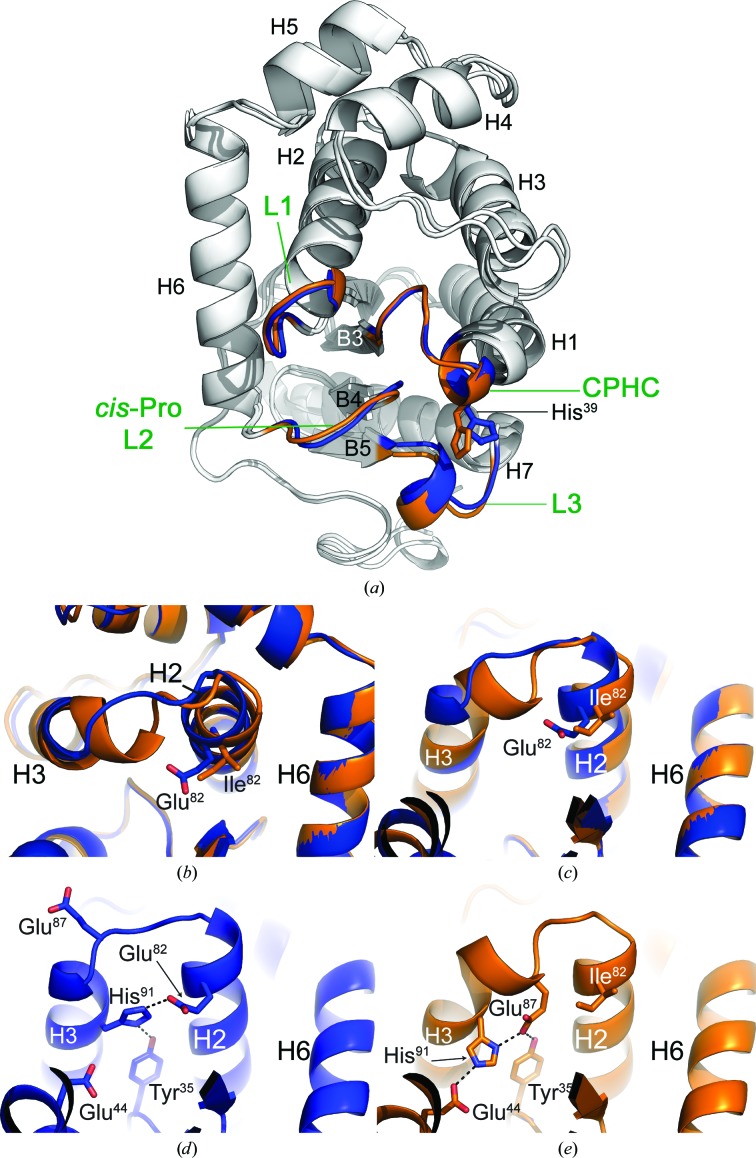
WT and E82I PaDsbA1 have a high degree of global structural equivalence with localized conformational differences. (*a*) Structural superposition of E82I PaDsbA1 (global superposition over all equivalent C^α^ atoms) on WT PaDsbA1 (PDB entry 3h93) shows a high similarity (r.m.s.d. of 0.94 Å between 180 equivalent C^α^ atoms). WT and E82I PaDsbA1 are coloured blue and orange, respectively, throughout. The connecting α-helix H6 in both WT and E82I PaDsbA1 overlaps precisely, and the thioredoxin domain also superimposes well. The main differences between the two structures localize primarily in the H2–H3 turn and N-terminal region of H3. The catalytic surface loops (CHPC active site, L1, *cis*-Pro L2 and L3) are highlighted (blue and orange for WT and E82I PaDsbA1, respectively). The side chain of active-site His39 that undergoes rotation in crystals 2 and 3 relative to crystal 1 and WT PaDsbA1 is shown in stick representation. (*b*, *c*) Close-up view of the H2–H3 turn and N-terminal region of H3. The view in (*c*) is rotated 180° around the *y* axis relative to (*a*) and that in (*b*) is rotated 45° around the *x* axis relative to (*c*). The E82I substitution disrupts the hydrogen-bonding pattern in this region, which introduces a kink and a partial helical turn to the N-terminal part of H3 in the E82I structure relative to the wild type. (*d*) Hydrogen-bonding pattern in the H2–H3 region of PaDsbA1. The side chain of His91 is hydrogen-bonded to the side chains of Glu82 and Tyr35. (*e*) Hydrogen-bonding pattern in the H2–H3 region of E82I PaDsbA1. Mutation of Glu82 to Ile results in the reorientation of His91, Glu87 and Glu44. In the E82I variant protein the side chain of His91 is connected by hydrogen bonds to the side chains of Glu87 and Glu44. (*d*) and (*e*) are orientated as in (*c*).

Superposition of WT and E82I PaDsbA1 indicates a high degree of global structural similarity (r.m.s.d. of 0.94 Å between 180 equivalent C^α^ atoms; Fig. 2 ▸ *a*). Importantly for subsequent fragment-based lead discovery projects, the positioning and conformation of the active-site surface loops is unaltered (Fig. 2 ▸ *a*).

As a direct result of the engineered amino-acid substitution, the WT PaDsbA1 and E82I PaDsbA1 structures differ in the conformation of their H2–H3 connecting loop (Fig. 2 ▸). Relative to WT PaDsbA1, a rearranged network of hydrogen bonding in E82I PaDsbA1 maintains the stabilization of particular H3 side chains but introduces both a kink and an additional partial helical turn to the N-terminal region of H3 (Figs. 2 ▸ *b* and 2 ▸ *c*). Specifically, in WT PaDsbA1 Glu82 forms a side-chain-mediated hydrogen bond to the N^∊2^ atom of the imidazole ring of His91. WT PaDsbA1 His91 is also involved in an additional side-chain contact (*via* its N^δ1^ atom) to the hydroxyl group of the neighbouring Tyr35 (Fig. 2 ▸ *d*). In E82I PaDsbA1, where the nonpolar aliphatic Ile side chain replaces Glu82, this Glu82-specific stabilization of His91 is lost, and His91 is instead stabilized by side-chain-mediated hydrogen bonds to Glu44 and Glu87 (Fig. 2 ▸ *e*). Furthermore, the side chain of His91 is flipped relative to its orientation in the wild-type protein (Figs. 2 ▸ *d* and 2 ▸ *e*). Glu44 is similarly positioned in both proteins, but in contrast Glu87 undergoes a significant repositioning in the E82I PaDsbA1 variant to mediate contact with His91; in this new orientation Glu87 is also suitably positioned to recapitulate a hydrogen bond to the Tyr35 hydroxyl group previously mediated by His91 N^δ1^. The overall effect of this altered hydrogen-bond network and the repositioning of Glu87 (Figs. 2 ▸ *d* and 2 ▸ *e*) is the introduction of a partial helical turn in H3 as a new main-chain helical hydrogen bond forms between His91 and Glu87. This N-terminal section of H3 forms part of the boundary of a groove on the posterior face of PaDsbA1 (Shouldice *et al.*, 2010 ▸); comparison of WT and E82I PaDsbA1 indicate that the posterior groove of both proteins is essentially unchanged.

### 3.3. E82I PaDsbA1 exhibits wild-type protein activity and redox character   

**Figure 3:**
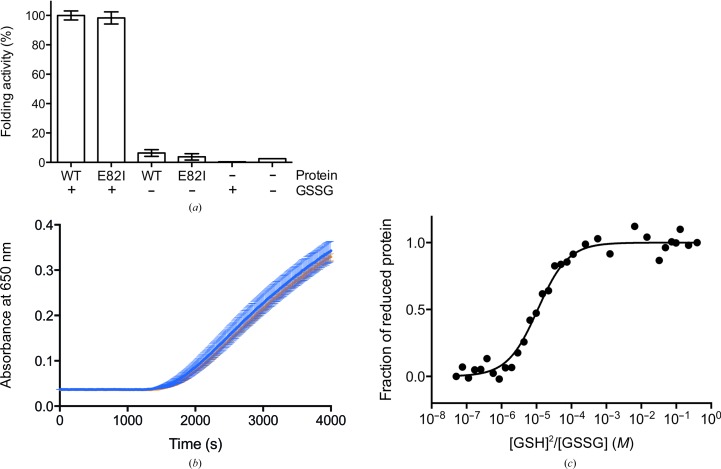
E82I PaDsbA1 exhibits wild-type protein activity and redox character. (*a*) WT and E82I PaDsbA1, in the presence of 2 m*M* oxidized glutathione, display a similar rate of oxidation of a fluorescently labelled peptide substrate. Reactions lacking glutathione, a protein catalyst or containing only buffer display negligible activity. The mean and SEM of four (WT) and three (E82I) independent biological replicates are plotted. For GSSG and buffer-only controls *n* = 1. (*b*) The disulfide reductase activity of WT and E82I PaDsbA1 was assessed using an insulin-reduction assay. WT (blue) and E82I (orange) PaDsbA1 catalyze the reduction of insulin in the presence of DTT to a similar extent. The mean and SEM of three independent biological replicates are plotted. (*c*) The fraction of oxidized and reduced E82I PaDsbA1 after equilibrium in redox buffers containing varying ratios of reduced glutathione:oxidized glutathione at pH 7.0 and 310 K was determined from the redox state-dependent fluorescence of PaDsbA1. These data were used to calculate *K*_eq_ and subsequently the redox potential. Representative data of a single experiment of six performed are shown, plotted on a semi-log graph using a base 10 logarithmic scale for the *x* axis and a linear scale for the *y* axis. For final redox potential determination, mean and SEM were calculated (*n* = 6).

DsbA proteins catalyze the oxidation of the thiol groups of cysteine residues to form stabilizing disulfide bonds. We measured the redox properties of the E82I PaDsbA1 variant and compared them with those of WT PaDsbA1 to determine whether or not the introduced mutation altered the ability of the protein to catalyze disulfide formation. Crucially, the E82I substitution does not affect protein function or redox character (Fig. 3 ▸). E82I PaDsbA1 has essentially identical activity to WT PaDsbA1 in both an *in vitro* peptide substrate oxidation assay (Fig. 3 ▸ *a*) and a classical insulin-reduction assay (Fig. 3 ▸ *b*). The E82I PaDsbA1 redox potential is unchanged [*K*_eq_ with glutathione of 1.19 ± 0.11 × 10^−5^ *M* (Fig. 3 ▸ *c*) relative to 9.96 ± 0.88 ×10^−6^ *M* for WT PaDsbA]. This equates to redox potentials of −92 and −94 mV, respectively.

### 3.4. Crystal structures of E82I PaDsbA1 with three cryoprotectants   

During the initial characterization of the E82I PaDsbA1 variant crystals, we explored a number of cryoprotectants to identify which was optimal for crystal cryocooling. In all, we determined the crystal structures of E82I PaDsbA1 cryoprotected with 40% PEG 400 (crystal 1, Table 1 ▸, PDB entry 4zl7), 20% glycerol (crystal 2, Table 1 ▸, PDB entry 4zl8) and 20% ethylene glycol (crystal 3, Table 1 ▸, PDB entry 4zl9). All three structures were determined at high resolution (in the range 1.4–1.9 Å) and were of comparable quality, although we note that the glycerol-cryoprotected crystals yielded the highest resolution diffraction data (Table 1 ▸).

#### 3.4.1. E82I PaDsbA1 can bind PEG molecules on its noncatalytic surface   

**Figure 4:**
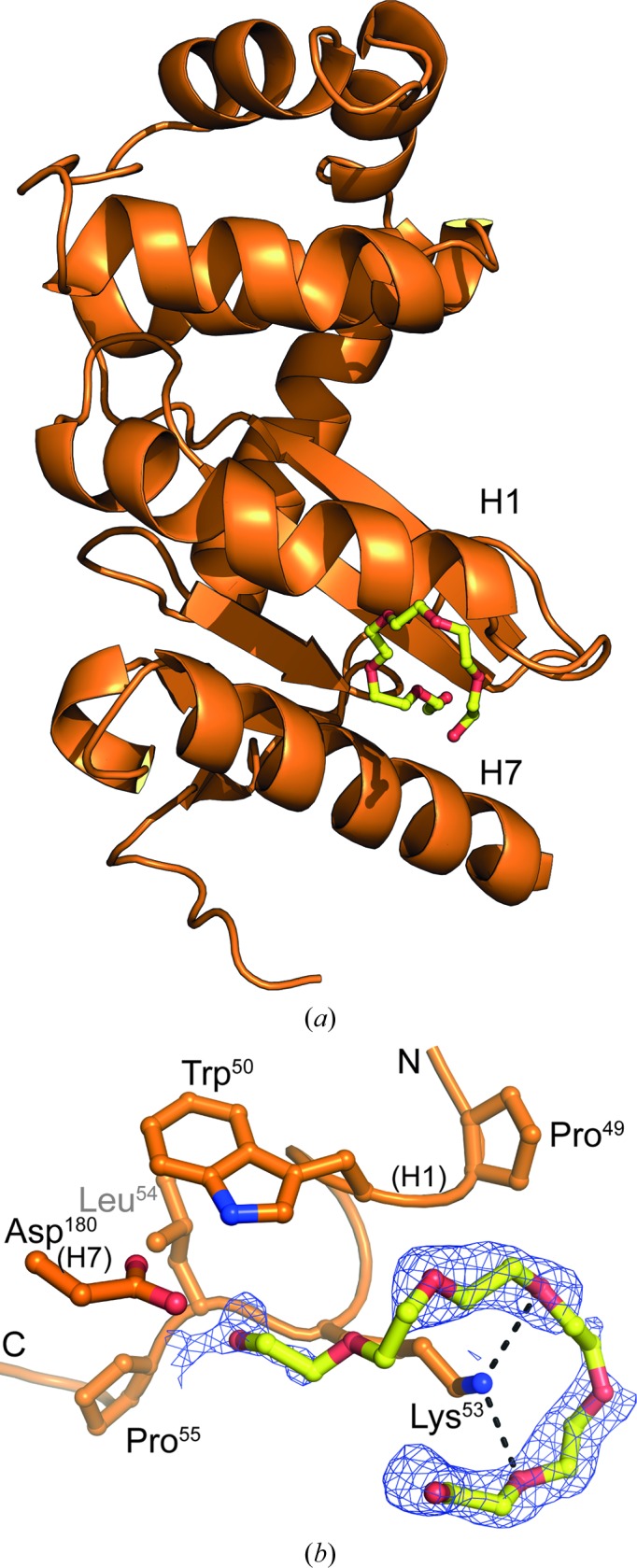
E82I PaDsbA1 binds a molecule of PEG 400 on its noncatalytic surface. (*a*) A PEG 400 molecule (yellow sticks) binds at the C-terminal end of H1, making hydrogen-bond interactions with the side chain of Lys53 (*b*). The OMIT map for PEG 400 is shown (*F*_o_ − *F*_c_ map contoured at 3σ obtained after refinement of the model in which the PEG 400 molecule was omitted).

A molecule of hexaethylene glycol (polyethylene glycol, PEG 400), which was used as a cryoprotectant during crystal cryocooling, is found in the electron-density map of E82I PaDsbA1 crystal 1. PEG 400 is bound at the C-terminal end of H1 (Fig. 4 ▸ *a*). The molecule adopts a horseshoe configuration encircling the side chain of Lys53 (Fig. 4 ▸ *b*). Electron density is sharply resolved for the majority of the molecule, where it is in closest proximity to Lys53, but is poorly resolved for the first two ethylene glycol monomer units of the molecule (Fig. 4 ▸ *b*); accordingly, atoms O1–C5 inclusive have elevated *B* factors with respect to the rest of the non-H atoms of the bound PEG 400. Two O atoms of PEG 400 make hydrogen-bonded contacts to Lys53 NZ: O10 and O16 (distances of 2.87 and 3.05 Å, respectively). PEG 400 makes additional contacts to Pro49, Trp50, Leu54 and Pro55 of H1 and Asp180 of H7.

We note that PEG 400 binds in a similar vicinity to that of a glycerol molecule (Glyc-3) resolved in the structure of WT PaDsbA1 (Shouldice *et al.*, 2010 ▸); in this structure Glyc-3 is positioned towards the C-terminal end of H1 between H1 and H7 within hydrogen-bonding distance of the side chains of H7 residues Asp180 and Arg187. As noted previously, this area is rich in acidic and basic residues (Shouldice *et al.*, 2010 ▸), and in crystal 3 (current study) a further PEG molecule from the crystallization condition can also be resolved near Asp187 and Glu184 of H7.

#### 3.4.2. E82I PaDsbA1 binds several small molecules at its active site   

E82I PaDsbA1 can also bind several small molecules directly on the catalytic face of the protein. In the crystal structures of the E82I PaDsbA1 variant cryocooled with either glycerol (crystal 2) or ethylene glycol (crystal 3), His39 in the active site makes electrostatic and hydrogen-bond contacts with a molecule of the respective cryoprotectant and also a molecule of 2-(*N*-morpholino)ethanesulfonic acid (MES). MES, which is presumably derived from the crystallization condition common to all three crystals, is not resolved in crystal 1, where His39 makes no apparent contacts to a component of the mother liquor or cryoprotectant.

**Figure 5:**
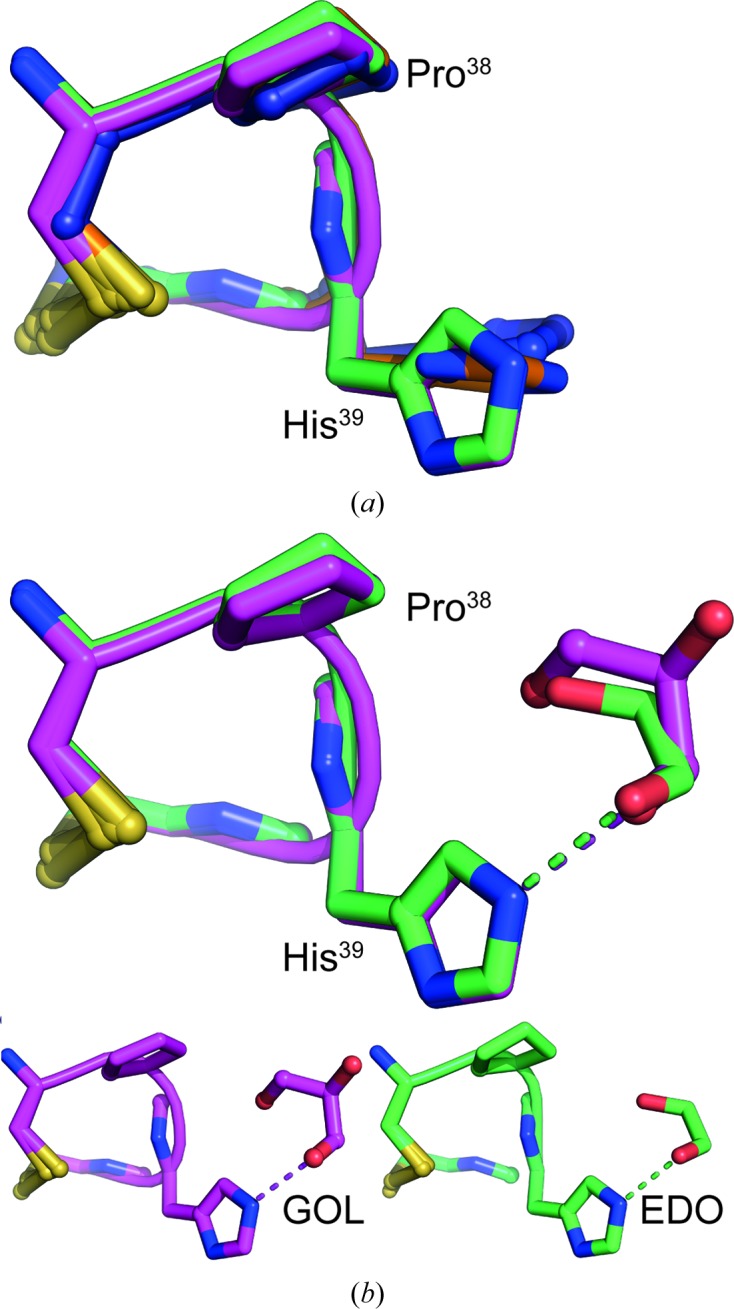
Rotation of the His39 side chain in response to small-molecule binding. (*a*) In the presence of glycerol (magenta) or ethylene glycol (green), the His39 side chain is rotated relative to its position in the crystal structures of either WT PaDsbA1 (blue) or E82I PaDsbA1 (orange) in the absence of a small molecule at the active site. (*b*) Glycerol (GOL) and ethylene glycol (EDO) bind at the same site in E82I PaDsbA1 and make equivalent hydrogen-bond contacts to His39.

In crystals 2 and 3, the orientation of the His39 imidazole ring is rotated relative to its position in the WT PaDsbA1 and PEG 400 cryocooled structures (crystal 1, present work; Fig. 5 ▸). It has been observed previously that the active-site histidine in *E. coli* DsbA can adopt a number of positions depending on the crystal form and the protein redox state (Guddat *et al.*, 1998 ▸). We suggest that in this case the observed reorientation of His39 is a direct consequence of glycerol or ethylene glycol binding, and that this in turn brings His39 into an orientation that is permissive for a stable interaction with MES.

**Figure 6:**
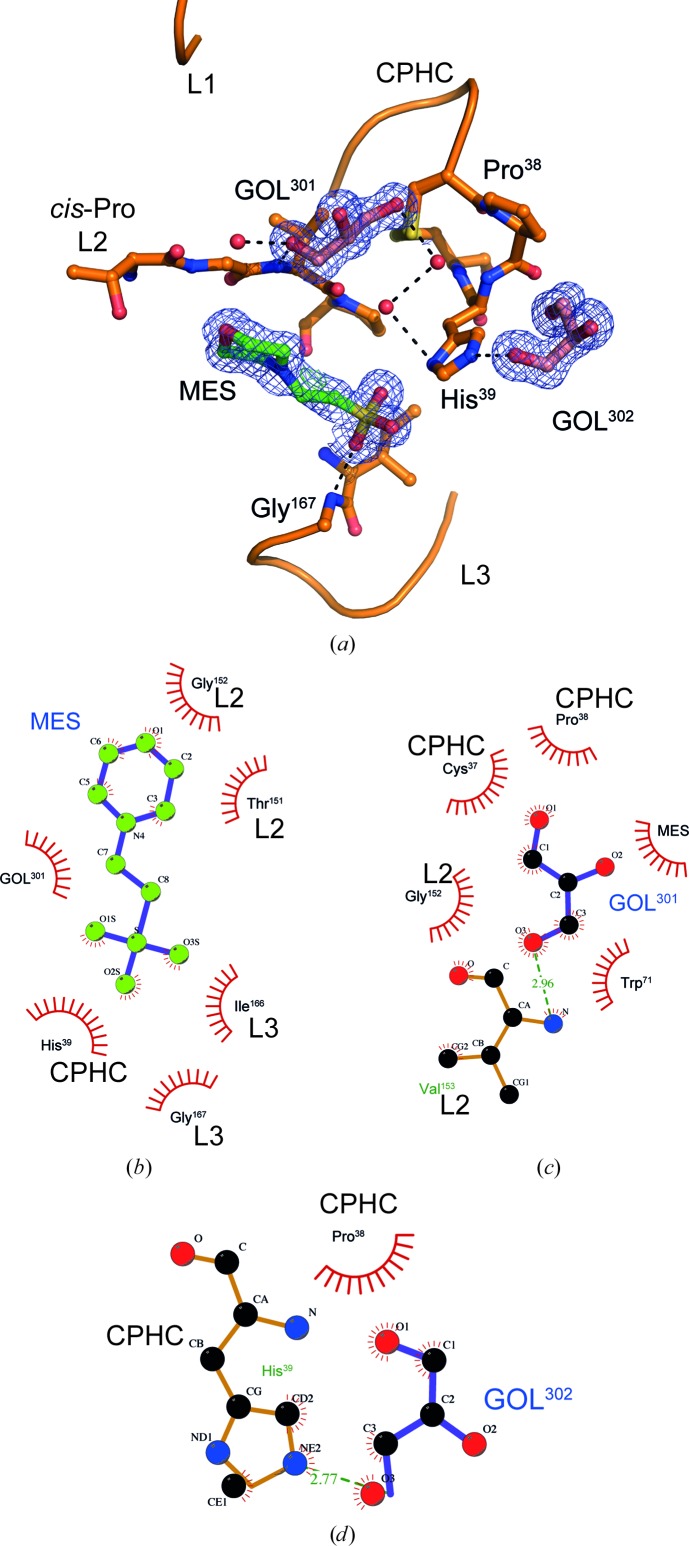
E82I PaDsbA1 binds several small molecules directly at its active site. (*a*) Three small molecules, MES (green) and glycerol (GOL; pink), are clearly resolved in the electron-density map of crystal 2 and make hydrogen-bond interactions with the CPHC active site and loops of the PaDsbA1 active-site surface (L1 linking β3 and H2, *cis*-Pro L2 linking H6 and β4, and L3 linking β5 and H7). The 2*F*_o_ − *F*_c_ electron-density maps for each molecule were generated from calculated phases using *phenix.maps* and are shown contoured at 1.0σ. The maps are shown within a 1 Å radius of each atom of MES or glycerol. (*b*) *LIGPLOT* representation of the environment of MES and (*c*, *d*) neighbouring glycerols in crystal 2.

The bound molecules of MES and cryoprotectant (glycerol or ethylene glycol) are each involved in numerous side-chain-mediated and main-chain-mediated hydrogen-bond inter­actions with residues of the active-site surface loops (CPHC loop, *cis*-Pro L2 and L3; Fig. 6 ▸). In crystal 2, three molecules (two glycerols and one MES) decorate the active-site loops, variously contacting Cys37, Pro38 and His39 (CPHC), Thr151, Gly152 and Val163 (cisPro L2), and Ile166 and Gly167 (L3) (Figs. 6 ▸ *b*, 6 ▸ *c* and 6 ▸ *d*). Together, this provides a serendipitous proof of principle that in the engineered E82I PaDsbA1 variant the active site is available to small molecules that are both present during crystallization (*i.e.* MES) and introduced *via* soaking (*i.e.* cryoprotectant glycerol).

## 4. Conclusion   

*P. aeruginosa* is a common cause of hospital-acquired infections (Rosenthal *et al.*, 2012 ▸). In the USA, 13% of severe nosocomial *P. aeruginosa* infections are multidrug-resistant, meaning that all or nearly all currently available antibiotics are ineffective as treatments (Centers for Disease Control and Prevention, 2013 ▸). ‘Prompt and sustained action’ including the development of new drugs is required to counter, and prevent the escalation of, the threat to public health (Centers for Disease Control and Prevention, 2013 ▸). DsbA is essential for multiple facets of *P. aeruginosa* virulence and is a highly promising target for new drug development. DsbA inhibitors would disrupt bacterial virulence rather than growth, serving as potent anti-infectives whilst exerting a reduced selection pressure to develop resistance (Heras *et al.*, 2015 ▸).

WT PaDsbA1 did not give crystals that were amenable for use in a fragment-screening approach. Here, we have generated a variant of PaDsbA1 to disrupt an active-site residue-mediated crystal contact. Whilst there are localized conformational changes to the N-terminal end of H3 of the variant protein E82I PaDsbA1, the active-site surface of the protein (CPHC, L1, *cis*-Pro L2 and L3) is unchanged, and crucially the protein activity and redox character are essentially identical to those of the wild type. In the identified crystal form of E82I PaDsbA1, the active-site region can simultaneously bind multiple ligands derived from the crystallization condition and cryoprotectant solution, indicating that the new crystal form is suitable for use in subsequent FBLD campaigns to characterize small-molecule binders of this important mediator of bacterial virulence.

In the early stages of fragment-based lead discovery campaigns, hit fragments typically exhibit low-affinity binding to their target. This presents an inherent challenge to obtaining protein–fragment crystal structures, in which a high occupancy of fragment in the crystal at the binding site is essential to resolve the corresponding electron density. It follows that to maximize the chances of successful soaking experiments one should avoid the potential for competition from other molecules that may also bind at the target site in the crystal. For this reason, we suggest that crystal 1 is the most suitable for subsequent structural and fragment-based lead discovery experiments, as either glycerol or ethylene glycol may directly or indirectly compete for fragment binding in this region.

## Supplementary Material

PDB reference: E82I PaDsbA1, 4zl7

PDB reference: 4zl8

PDB reference: 4zl9

## Figures and Tables

**Table 1:** Data collection and processing

	Crystal 1	Crystal 2	Crystal 3
PDB code	4zl7	4zl8	4zl9
Cryoprotectant	40% PEG 400	20% glycerol	20% ethylene glycol
Wavelength (Å)	0.95370	0.95370	0.95370
Resolution range (Å)	41.26–1.92 (2.03–1.92)	34.91–1.40 (1.45–1.40)	41.66–1.70 (1.76–1.70)
Space group	*P*2_1_	*P*2_1_	*P*2_1_
Unit-cell parameters (Å, °)	*a* = 35.3, *b* = 62.3, *c* = 41.6, α = 90.0, β = 97.6, γ = 90.0	*a* = 35.3, *b* = 63.7, *c* = 42.0, α = 90.0, β = 98.3, γ = 90.0	*a* = 35.4, *b* = 62.9, *c* = 42.1, α = 90.0, β = 98.2, γ = 90.0
*R*_merge_	0.081 (0.405)	0.080 (0.361)	0.110 (0.763)
Total No. of observations	47377 (6746)	132345 (18730)	72341 (10588)
Total No. unique	13668 (1981)	36539 (5334)	19942 (2863)
Mean *I*/σ(*I*)	9.9 (2.6)	9.7 (3.2)	7.8 (1.9)
Completeness (%)	99.6 (99.5)	99.8 (99.9)	99.1 (98.2)
Multiplicity	3.5 (3.4)	3.6 (3.5)	3.6 (3.7)

**Table 2:** Refinement and model quality Values in parentheses are for the highest resolution shell.

	Crystal 1	Crystal 2	Crystal 3
*R* factor (%)	15.90 (19.76)	14.60 (20.57)	15.92 (23.32)
*R*_free_ † (%)	20.21 (23.92)	15.94 (24.36)	20.07 (27.03)
No. of atoms			
Total	3074	3284	3227
Macromolecules	1475	1494	1476
Ligands	19	36	29
Water	111	205	222
No. of protein residues	185	187	187
R.m.s.d., bonds (Å)	0.012	0.009	0.010
R.m.s.d., angles (°)	1.34	1.29	1.27
Ramachandran favoured (%)	98	99	99
Ramachandran outliers (%)	0	0	0
*MolProbity* clashscore	5.4	1.32	3.67
Percentile‡	96th [*N* = 743, 1.922 ± 0.25 Å]	99th [*N* = 453, 1.395 ± 0.25 Å]	97th [*N* = 819, 1.700 ± 0.25 Å]
*MolProbity* overall score	1.29	0.85	1.16
Percentile‡	99th [*N* = 11840, 1.922 ± 0.25 Å]	100th [*N* = 3078, 1.395 ± 0.25 Å]	99th [*N* = 9248, 1.700 ± 0.25 Å]
Average *B* factor (Å^2^)			
Protein	20.7	17.6	17.7
Ligand	45.3	24.1	41.1
Solvent	27.7	28.7	26.8

† *R*_free_ was calculated with 5% of the data set.

‡ The 100th percentile is the best among structures of comparable resolution; the 0th percentile is the worst. For the *MolProbity* clashscore the comparative set of structures was selected in 2004; that for the *MolProbity* score was selected in 2006.

**Table 3:** Number of active-site surface-loop residues involved in crystal-packing interactions in WT and E82I PaDsbA1 crystals Crystal contacts were identified using *CONTACT* within the *CCP*4 suite (Winn *et al.*, 2011 ▸), searching for symmetry contacts ≤4 Å to protein atoms only. Atoms within hydrogen-bond contact distance of a suitable hydrogen-bond donor/acceptor are shown. The key His39 to Glu82_sym_ contact in WT PaDsbA1 is italicized.

	WT PaDsbA1			E82I PaDsbA1		
	No. of residues	Residue name	Hydrogen-bond contact	No. of residues	Residue name	Hydrogen-bond contact
CHPC	3	Trp34	—	3	Trp34	—
		Cys37	—		Gly36	—
		*His39 *	*Glu82_sym_ (2.88 Å)*		Pro38	—
Loop 1	1	Phe67	—	2	Gly69	—
					Ile70	—
*cis*-Pro loop (loop 2)	4	Val150	—	—	—	—
		Thr151	Thr128_sym_ (3.25 Å)		—	—
		Gly152	—		—	—
		Val153	Ser83_sym_ (3.25 Å)		—	—
Loop 3	3	Asp165	—	—	—	—
		Gly167	—		—	—
		Ser168	—		—	—
